# Fulminant hepatitis during treatment for per-extensively drug-resistant tuberculosis: A case report and call for improved patient monitoring

**DOI:** 10.1097/MD.0000000000045893

**Published:** 2025-11-21

**Authors:** Tian Zheng, Aierkenjiang Malipati, Shanshan Guo, Wenlong Guan, He Yang, Hongxia An, Abuduwaili Rouzihan, Abudushalamu Rukeyamu, Abudureheman Kaliman

**Affiliations:** a The First Department of Tuberculosis, The Sixth People’s Hospital of Xinjiang Uygur Autonomous Region, Urumqi, Xinjiang, China.

**Keywords:** combination therapy, drug, extensively drug, fulminant hepatitis, induced liver injury, multidrug, multidrug, per, resistant tuberculosis, resistant tuberculosis

## Abstract

**Rationale:**

Drug therapy is the most effective therapy for tuberculosis; however, it can also lead to a common and serious adverse reaction known as antituberculosis drug–induced liver injury. The incidence of antituberculosis drug–induced liver injury ranges from 2.0% to 28.0%.

**Patient concerns:**

Here, we report the successful treatment of drug-induced fulminant hepatitis in a 19-year-old patient with extensively drug-resistant tuberculosis.

**Diagnoses:**

The patient was treated with a combination of bedaquiline, linezolid, cycloserine, clofazimine, amikacin, ethambutol, and pyrazinamide. This combination was selected because the patient showed resistance to rifampicin, isoniazid, and fluoroquinolones in a drug resistance test. However, for personal reasons, the patient did not follow the doctor’s advice and developed fulminant hepatitis induced by antituberculosis drugs.

**Interventions:**

Antituberculosis drugs were immediately discontinued, and the patient received clinical treatment for liver protection, jaundice elimination, artificial liver support, and infusion of fibrinogen and prothrombin complex.

**Outcomes:**

Gradually, the patient’s liver function returned to normal.

**Lessons:**

This case report highlights the importance of regular liver function monitoring during antituberculosis therapy to ensure patient safety.

## 1. Introduction

Tuberculosis continues to pose a significant threat to public health and is one of the leading causes of death worldwide. In 2023, it is estimated that 10.8 million people will be affected by tuberculosis and that 1.25 million people will die from the disease.^[1]^ The emergence of antibiotic resistance is a growing and concerning issue that has led drug-resistant tuberculosis and its diagnosis and treatment to become a major focus of attention.^[2,3]^ Multidrug-resistant tuberculosis (MDR-TB) is caused by *Mycobacterium tuberculosis*, which is resistant to at least 2 first-line drugs: isoniazid (INH) and rifampicin (Rif).^[4]^ Pre-extensively drug-resistant tuberculosis (pre-XDR-TB) is resistant to quinolones or second-line injection antituberculosis drugs, in addition to being multidrug-resistant.^[5,6]^ Globally, data from 105 countries and regions show that the average proportion of *M. tuberculosis* strains in MDR-TB that are resistant to fluoroquinolones is 20.1%.^[4]^ Patients with MDR/RR-TB or pre-XDR-TB require longer treatment regimens and are at higher risk of adverse events than those with drug-sensitive tuberculosis, depending on the second-line drugs used.^[7,8]^

Among the drugs approved for the treatment of tuberculosis, INH, Rif, ethambutol (Emb), and pyrazinamide (Pza) are considered first-line drugs and form the core of standard treatment regimen.^[9]^ Furthermore, bedaquiline (Bdq), linezolid (Lzd), clofazimine (Cfz), cycloserine (Cs), and amikacin (Am) are recommended by the WHO for the long-term treatment of MDR-TB.^[7,10]^ Although these treatments are effective against active tuberculosis, they are also associated with numerous adverse drug reactions, which pose a major challenge to treatment completion.^[11,12]^ One of the most common adverse reactions is antituberculosis drug-induced liver injury (ATLI), which can hinder patient adherence to treatment and negatively impact treatment outcomes.^[12–14]^ This report presents a case of acute ATLI in a patient with pre-XDR-TB who was administered a combination of Bdq, Lzd, Cs, Cfz, Am, Emb, and Pza.

## 2. Case presentation

### 2.1. Patient case information

A 19-year-old male patient was admitted to our hospital on February 13, 2023, due to persistent cough, expectoration, fever, and fatigue that had been ongoing for 2 months. The patient denied any history of smoking, drinking, drug use, or night travel. He was unmarried, had no children, and had no significant medical or family history. The symptoms before admission are described in the supplementary materials (File S1, Supplemental Digital Content, https://links.lww.com/MD/Q655). A sputum acid-fast bacilli test conducted at a hospital in Urumqi before admission to our hospital revealed a class 3 + result, which was preliminarily diagnosed as tuberculosis.

### 2.2. Physical examination on admission

His body temperature was 36.8 °C, his weight was 70 kg, his pulse was 96 beats/min, his breathing frequency was 21 beats/min, and his blood pressures were 114/60 mm Hg. Both lungs had coarse breathing sounds but dry and wet rales were not heard. Additionally, no obvious abnormalities were observed on cardiac examination.

### 2.3. Auxiliary examination after admission

**Figure 1.**
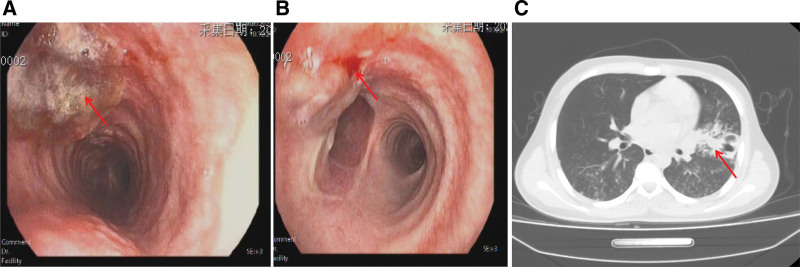
Electronic bronchoscopy and lung CT images of the patient revealed significant abnormalities. (A) The bronchial mucosa appeared hyperemic and edematous, with evidence of caseous necrosis on the left side of the trachea. The surrounding mucosa also appeared uneven (indicated by the red arrow). (B) Further examination showed extensive cheese-like attachment along the carina to the left upper lobe, which resulted in blockage of the tongue lobe opening and narrowing of the proper branch opening. Additionally, there was evidence of cheese-like attachment and a large amount of purulent and jelly-like secretions overflowing (indicated by the red arrow). (C) The lingual segment of the patient’s left lung appeared consolidated, with scattered spots, strips, and nodular density-increased shadows (indicated by the red arrow).

The detection methods for physiological and biochemical indices in patient blood are described in supplementary materials (File S2, Supplemental Digital Content, https://links.lww.com/MD/Q655). The patient’s blood tests showed normal results, whereas his serum erythrocyte sedimentation rate (ESR), fibrinogen, D-dimer, total bilirubin (TBIL), direct bilirubin (DBIL), γ-glutamyl transferase, alkaline phosphatase (ALP), lactatedehydrogenase (LDH), and total bile acid levels were higher than normal (Table 1). His kidney function was normal, a sputum smear for *M. tuberculosis* showed 2 + levels of acid-fast bacilli, and the molecular test result for *M. tuberculosis* was positive. Type B ultrasonography revealed multiple lymphadenopathies in the right side of the neck and chronic cholecystitis. Electronic bronchoscopy and lung CT images showed that the bronchial mucosa was hyperemic and edematous, with evidence of caseous necrosis on the left side of the trachea, and the surrounding mucosa was uneven (Fig. 1A). Further examination revealed extensive cheese-like attachment along the carina to the left upper lobe, which resulted in blockage of the tongue lobe opening and narrowing of the proper branch opening. Additionally, there was evidence of a cheese-like attachment and a large amount of overflowing purulent and jelly-like secretions (Fig. 1B). The lingual segment of the patient’s left lung appeared consolidated with scattered spots, strips, and nodular density-increased shadows (Fig. 1C). Blood gas analysis, lung function tests, and electrocardiography results were normal, and the tests for hepatitis and other infectious diseases yielded negative results. Electronic bronchoscopy revealed bronchial tuberculosis with inflammatory infiltrates, and bronchial aspirates also showed 2 + levels of acid-fast bacilli. The Xpert MTB/RIF assay indicated that the strain was resistant to Rif, and gene probe assays revealed mutations in the resistance genes Rif (*rpoB*), Inh *(katG*), and fluoroquinolone (*gyrA*). Therefore, the patient was diagnosed with secondary left bronchial Pre-XDR-TB (ulcer necrosis) and right cervical lymph node tuberculosis.

**Table 1:** Blood indices of the patient at admission and during antituberculosis drug-induced liver injury.

Stage	Blood index	Value	Normal value
Admission	ESR (mm/h)	83	<15
	Fibrinogen (g/L)	5.92	2–4
	D-Dimer (mg/L)	2.10	<0.5
	TBIL (μmol/L)	28.1	5.13–22.24
	DBIL (μmol/L)	17.4	0–6.8
	GGT (U/L)	146.8	<50
	ALP (U/L)	131.4	53–128
	LDH (U/L)	269.6	109–245
	TBA (μmol/L)	269	0.1–10.0
Antituberculosis drug-induced liver injury	WBC (×10^9^ cells/L)	14.25	4.0–10.0
	Neutrophil count (×10^9^ cells/L)	11.77	1.8–6.3
	PT (s)	51	11.0–13.0
	Prothrombin time activity (%)	12.80	75–100
	Fibrinogen (g/L)	1.19	2–4
	D-Dimer (mg/L)	0.77	<0.5
	TBIL (μmol/L)	522.43	5.13–22.24
	DBIL (μmol/L)	348.07	0–6.8
	ALT (U/L)	354	<35
	AST (U/L)	377	<40
	GGT (U/L)	49.58	<50
	ALP (U/L)	192.91	53–128
	LDH (U/L)	386.63	109–245
	TBA (μmol/L)	328.57	0.1–10.0
	Creatinine (μmol/L)	49.21	44–132
	BUN (mmol/L)	0.97	3.2–7.1
	Uric acid (μmol/L)	178.36	150–416

ALP = alkaline phosphatase, ALT = alanine aminotransferase, AST = aspartate aminotransferase, BUN = blood urea nitrogen, DBIL = direct bilirubin, ESR = erythrocyte sedi-mentation rate, GGT = γ-Glutamyl transferase, LDH = lactatedehydrogenase, PT = prothrombin time, TBA = total bile acid, TBIL = total bilirubin, WBC = white blood cell count.

### 2.4. Treatment process after admission

On February 13th, the patient was prescribed a combination of medications for the treatment of tuberculosis. The treatment included oral administration of 400 mg/(day · time) of Bdq (taken as 200 mg/time with 3 times per week after 14 days), 600 mg/(day · time) of Lzd tablets, 100 mg/(day · time) of Cfz, 250 mg/time of Cs capsules with 50 mg/time of vitamin B6 (twice a day), 0.5 g/time of Pza tablets (3 times/day), and 0.75 g/(day · time) of Emb tablets, and intramuscular injection of 0.6 g/(day · time) of Am (0.2 g/piece). Additionally, the patient underwent electronic bronchoscopy every other week to monitor bronchial tuberculosis.

On March 26th, the patient was discharged on medication. The patient’s ESR was 37 mm/h, fibrinogen was 4.22 g/L, D-dimer was 1.27 mg/L, and liver and kidney function, electrolyte levels, and routine urine and stool tests were all normal before discharge.

However, after discharge, the patient did not follow the doctor’s advice to regularly check his liver and kidney functions or perform routine blood tests. By May 19th, the patient experienced abdominal pain, diarrhea, and yellow, shapeless stools. These symptoms were followed by general fatigue, loss of appetite, drowsiness, decreased urine output (300 mL/day) and occasional complete lack of urine output. The patient also gained 5 kg in 5 days. Consequently, the patient sought emergency treatment at our hospital on May 25th. The tests showed that the patient’s white blood cell count, neutrophil count, prothrombin time, D-dimer, TBIL, DBIL, alanine aminotransferase (ALT), aspartate aminotransferase (AST), ALP, LDH, and total bile acid levels were all higher than normal, whereas the prothrombin time activity and fibrinogen and blood urea nitrogen levels were lower than normal (Table 1). Electrocardiography revealed that the patient had a normal sinus rhythm, with an abnormal Q wave in the lower wall and a heart rate of 86 beats/min. After ruling out autoimmune, hereditary, metabolic, and infectious causes of the acute liver failure, the patient was diagnosed with acute ATLI. As a result, the patient immediately stopped taking antituberculosis medication and received treatment for liver protection, enzyme reduction, catharsis, 5 plasma exchanges, fibrinogen and prothrombin complex infusion, and nutritional support.

After receiving treatment, the patient’s symptoms gradually improved, and jaundice subsided. Reexamination of his liver function showed that his bilirubin and transaminase levels approached normal levels. Within 20 days, his PT activity recovered to 50%, which indicated the reversal of liver failure. All laboratory parameters returned to normal, and the patient was discharged on June 21. Following discharge, all related examinations revealed no abnormalities as advised by the physician.

**Figure 2.**
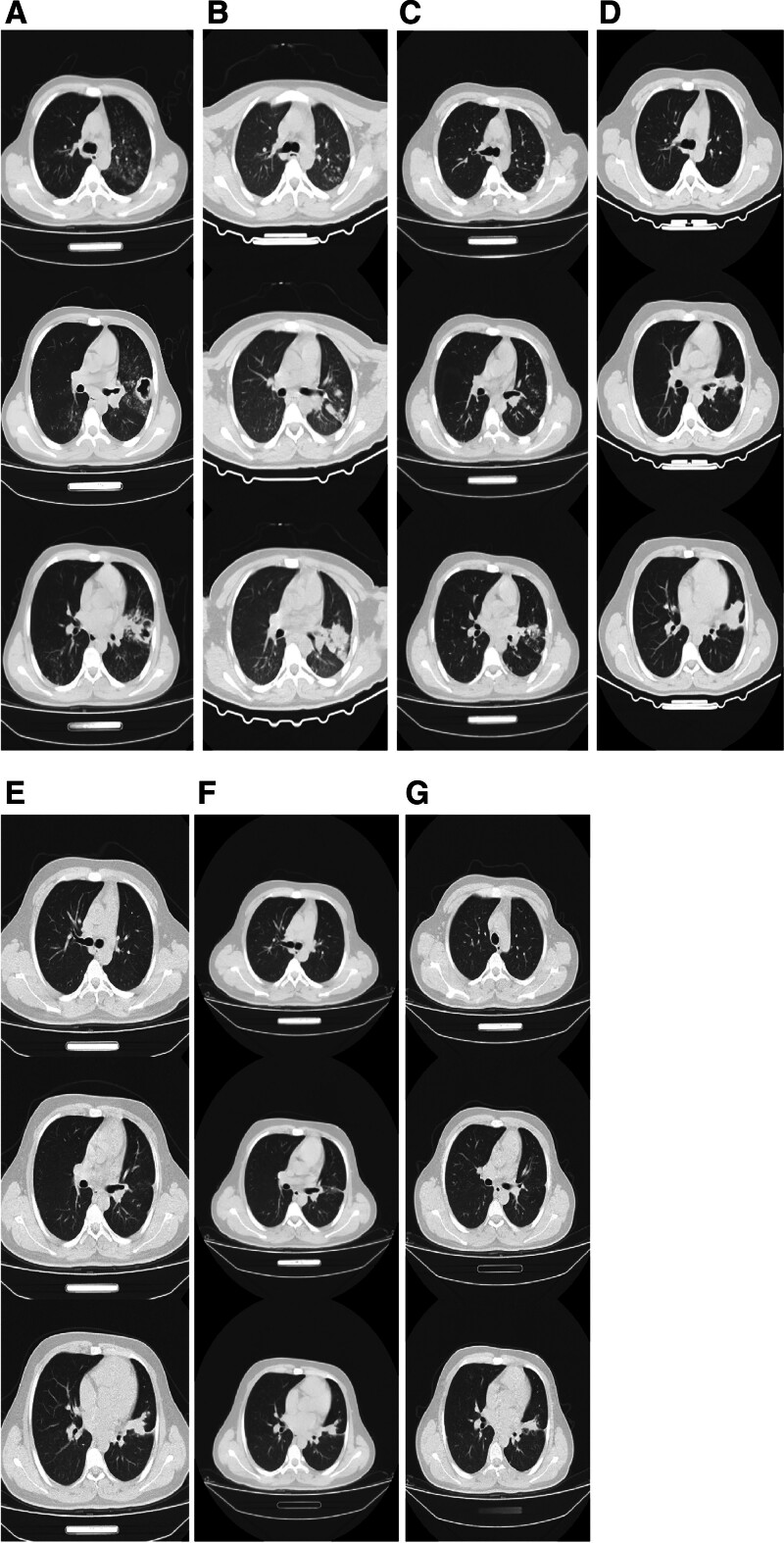
Progression of the patient’s chest CT images during treatment. The images were taken in (A) March 2023, (B) May 2023, (C) July 2023, (D) October 2023, (E) February 2024, (F) August 2024, and (G) December 2024.

On July 25^th^, the patient underwent reexamination. His TBIL was 26.01 μmol/L, DBIL was 16.30 μmol/L, and his transaminases were within normal range. His ESR was 46.00 mm/h, and there were no notable abnormalities in his routine blood tests or coagulation functions. Type B ultrasonography revealed thickening of the liver parenchyma, chronic cholecystitis, and splenomegaly. The patient also had multiple swollen lymph nodes on the right neck. The GeneXpert test for *M. tuberculosis* was positive, similar to the sputum smear. Bronchoscopy revealed left bronchial tuberculosis (scar stenosis), and a lung CT scan showed multiple translucent areas on the posterior wall of the lingual segment of the left lung, along with secondary dilatation of some bronchi. Both lungs showed scattered spots, cords, and shadows with increased nodular density. After discussion with general practitioners, the patient was orally administrated 600 mg/(day · time) of Lzd tablets, 200 mg/time of Bdq with 3 times per week, 100 mg/(day · time) of Cfz, 250 mg/time of Cs capsules with twice a day, and 1 g/(day · time) of Emb tablets to treat the progressing tuberculosis. The patient was regularly reviewed and underwent followups. By October 2024, the patient’s lesions had significantly improved, as evidenced by regular lung CT scans and negative tuberculosis culture, molecular detection, and sputum smear results (Fig. 2 and Table S1, Supplemental Digital Content, https://links.lww.com/MD/Q655).

The patient declared that he was very satisfied with the detection and treatment that he received and agreed with the doctor’s emphasis that he should strictly follow the doctor’s advice if he fell ill in the future.

## 3. Discussion

Several factors contribute to the discontinuation of tuberculosis treatment, the most significant being ATLI.^[13,15–17]^ Approximately 11.90% of liver injuries are caused by antituberculosis drugs,^[18]^ and clinical symptoms of ATLI include jaundice, nausea, vomiting, rash, and itching.^[19,20]^ Liver injury can result in tuberculosis treatment discontinuation, which can impact treatment effectiveness and increase the risk of drug resistance.^[15,17]^ Severe disease can lead to acute liver failure and death.^[17]^ A derivation cohort study that involved 3155 patients showed that 170 (5.4%) patients developed ATLI, of whom 27 (15.9%) developed jaundice, 9 (5.3%) developed acute liver failure, and 3 died.^[16]^ The investigators found that age, baseline AST level, previous TB treatment, and active drinking were risk factors for developing jaundice.^[16]^ Because the recommended duration of pulmonary tuberculosis therapy is 6 months or more,^[21–23]^ most treatments in China are usually completed by taking medicine at home, which presents obstacles to the management of liver health during treatment. Our results highlight the challenge of patient retention and adherence during long-term, toxic outpatient regimens for MDR/RR-TB. Although patients who take medicine at home should pay more attention to the health management of their liver, patient management systems must be robust enough to prevent patients from being lost to follow-up.

Among the first-line antituberculosis drugs, Pza is the most common cause of drug-induced liver damage, which typically results in hepatocellular liver damage.^[24,25]^ Shu et al.^[26]^ reported that the incidence of Pza-related DILI was 3.71/100 patients/month, which is higher than that of INH- or Rif-related DILI. The toxicity of Pza is dose-dependent, with high doses of 40 to 50 mg/kg, and long-term exposure is associated with DILI.^[27]^ However, our findings suggest that long-term severe DILI can still occur, even with a standard dose of Pza. In this case, the patient was diagnosed with pre-XDR-TB, and the treatment plan was determined by a drug-resistant tuberculosis expert group. However, the patient did not regularly monitor his liver and kidney function, as advised by the doctor during the antituberculosis treatment, which resulted in liver damage from hepatocyte injury. After receiving symptomatic treatment such as artificial liver support, the patient’s symptoms improved. As the patient’s liver function recovered, antituberculosis drugs were gradually reintroduced, and the patient was eventually cured.

Although patients with ATLI have been widely reported,^[14,28,29]^ and a recent study found that ferroptosis plays a crucial role in the development of DILI,^[30]^ how to assess the risk of ATLI before treatment still needs to be thoroughly studied. Notably, this is also an important issue that currently plagues the treatment of pulmonary tuberculosis. Additionally, because the patient was admitted to our hospital for examination and treatment after experiencing severe symptoms of liver damage, we did not know the developmental process of the liver damage before admission. Moreover, this study failed to address the issue of liver health monitoring during home treatment, although our findings definitively highlight the importance of liver health monitoring during treatment. Furthermore, patient management systems must be robust enough to prevent patients from being lost during the follow-up period of tuberculosis treatment.

## 4. Conclusion

Our results highlight the challenge of patient retention and adherence during long-term, toxic outpatient regimens for MDR/RR-TB. ATLI is a common adverse reaction to MDR/RR-TB treatments. Although patients who take medicine at home should pay more attention to the health management of their liver, patient management systems must be robust enough to prevent patients from being lost to follow-up.

## Acknowledgments

We would like thank Dr Jiajia Ni from Guangdong Meilikang Bio-Science Ltd., China for his assistance in the manuscript review and editing.

## Author contributions

**Conceptualization:** Aierkenjiang Malipati, Abudushalamu Rukeyamu, Abudureheman Kaliman.

**Data curation:** Aierkenjiang Malipati.

**Formal analysis:** Tian Zheng, Shanshan Guo, Wenlong Guan, He Yang, Hongxia An, Abuduwaili Rouzihan.

**Funding acquisition:** Abudushalamu Rukeyamu.

**Investigation:** Tian Zheng, Wenlong Guan, He Yang, Hongxia An, Abuduwaili Rouzihan.

**Methodology:** Aierkenjiang Malipati, Wenlong Guan, Abudushalamu Rukeyamu.

**Resources:** Abudushalamu Rukeyamu.

**Software:** Aierkenjiang Malipati.

**Supervision:** Abudureheman Kaliman.

**Writing – original draft:** Tian Zheng.

**Writing – review & editing:** Abudushalamu Rukeyamu.

## Supplementary Material


