# Supplementary material for: Fulminant hepatitis during treatment for per-extensively drug-resistant tuberculosis: A case report and call for improved patient monitoring
Source: Medicine (Baltimore). 2025 Nov 21;104(47):e45893. doi: 10.1097/MD.0000000000045893 (PMC12643652; doi:10.1097/MD.0000000000045893)
Supplement: Supplementary file 1 [file medi-104-e45893-s001.pdf]

File S1. The symptoms of the patient before admission.

In December 2022, the patient experienced occasional coughing with a small amount of yellow pus and phlegm. In the afternoon, he had fever with body temperature of 37.2 to 37.6 °C, accompanied by loss of appetite and fatigue. However, he did not pay much attention to his symptoms and they gradually worsened. By the end of January 2023, he began experiencing tremors in his hands, particularly on the right side, and night sweats. An MRI scan of his head at a local hospital showed no abnormalities. He then went to a hospital in Urumqi, where tuberculosis was suspected after a chest imaging examination. The sputum acid-fast bacilli test revealed a class 3+ result, and he was transferred to our hospital on February 13th, 2023.

File S2. Detection methods of blood physiological and biochemical indices.

The blood routine indices and CRP were determined using an automatic cell analyzer CAL800 (Mindray, Shenzhen, China). The coagulation D-dimer was determined using an automatic coagulation analyzer CS-5100 (Sysmex Europe, Germany). The procalcitonin was determined using an active immunity analyzer QD-S2000 (Vazyme Medical Technology, Nanjing, China). Blood gas analysis was conducted using an ABL90FLEX (Radiometer, Shanghai, China). The blood biochemical indices were determined using a BS-2800M automatic biochemical analyzer (Mindray, Shenzhen, China). Acid-fast staining of *M. tuberculosis* was conducted using the KPJ/TIR-800 system (Keruijie, Changsha, China). Molecular *M. tuberculosis* detection was conducted using the AMPLIFIED MTD kit (Bio-Meria, China) on a Deaou-IS48 constant temperature amplification fluorescence detector (Deaou, Guangzhou, China). GeneXpert (Cepheid, USA) detection was conducted on a GeneXpert real-time fluorescence PCR system GX-IV R2 (Cepheid, USA).

Table S1. Process of the patients' pathogenic *Mycobacterium tuberculosis* examination.

Detection data	Sputum smear	Molecular detection	Sputum tuberculosis smear
February, 2023	2+	+	Growth of *M. tuberculosis*: R-HRFQ
March, 2023	2+	+	Growth of *M. tuberculosis*: R-HRFQ
July, 2023	+	+	Unisolating *M. tuberculosis*
August, 2023	—	—	Unisolating *M. tuberculosis*
December, 2023	—	—	Unisolating *M. tuberculosis*
March, 2024	—	—	Unisolating *M. tuberculosis*
May, 2024	—	—	Unisolating *M. tuberculosis*
August, 2024	—	—	Unisolating *M. tuberculosis*
September, 2024	—	—	Unisolating *M. tuberculosis*
